# Policy responses to hepatitis C in the Nordic countries: Gaps and discrepant reporting in the Hep-Nordic study

**DOI:** 10.1371/journal.pone.0190146

**Published:** 2018-01-30

**Authors:** Kelly Safreed-Harmon, Kristina L. Hetherington, Soo Aleman, Hannu Alho, Olav Dalgard, Tove Frisch, Magnus Gottfredsson, Nina Weis, Jeffrey V. Lazarus, on behalf of the Hep-Nordic Study Group

**Affiliations:** 1 CHIP, Rigshospitalet, University of Copenhagen, Copenhagen, Denmark; 2 Barcelona Institute for Global Health (ISGlobal), Hospital Clínic, University of Barcelona, Barcelona, Spain; 3 Department of Infectious Diseases, Karolinska Institutet/Karolinska University Hospital, Stockholm, Sweden; 4 Abdominal Center, Helsinki University Hospital, Helsinki, Finland; 5 Department of Infectious Diseases, Akershus University Hospital, Lørenskog, Norway; 6 Riksföreningen Hepatit C, Månkarbo, Sweden; 7 Faculty of Medicine, School of Health Sciences, University of Iceland, Reykjavik, Iceland; 8 Department of Internal Medicine, Division of Infectious Diseases, Landspitali University Hospital, Reykjavik, Iceland; 9 Department of Infectious Diseases, Copenhagen University Hospital, Hvidovre, Denmark; 10 Department of Clinical Medicine, Faculty of Health and Medical Sciences, University of Copenhagen, Copenhagen, Denmark; Centers for Disease Control and Prevention, UNITED STATES

## Abstract

**Background and aims:**

In the Nordic countries (Denmark, Finland, Iceland, Norway, Sweden), the prevalence of chronic hepatitis C virus (HCV) infection is relatively low in the general population, but is much higher among people who inject drugs (PWID). We conducted an exploratory study to investigate the extent to which these countries have policies supporting key elements of the public health response that is necessary to achieve the global goal of eliminating HCV as a public health threat.

**Methods:**

Fourteen stakeholders representing government agencies, medical societies, and civil society organisations (CSOs) in the Nordic countries completed a cross-sectional online survey that included 21 policy questions related to national coordination, prevention, testing, linkage to care, and treatment. We summarised the findings in a descriptive analysis, and noted discrepant responses from stakeholders within the same country.

**Results:**

Stakeholders reported that three of the five study countries have national viral hepatitis strategies, while only Iceland has a national HCV elimination goal. The availability of harm reduction services varies, with opioid substitution therapy provided for the general population throughout all countries, but not needle and syringe programmes. No country has access to anonymous HCV testing in all parts of the country. National HCV treatment guidelines are available in all countries except Finland, and all countries provide publicly funded direct-acting antiviral treatment. Disagreement regarding policies was observed across countries, and CSOs were the stakeholder group that most frequently answered survey questions incorrectly.

**Conclusion:**

The Nordic region as a whole has not consistently expressed its commitment to tackling HCV, despite the existence of large HCV epidemics among PWID in these countries. Stakeholder alignment and an established elimination goal with an accompanying strategy and implementation plan should be recognised as the basis for coordinated national public health efforts to achieve HCV elimination in the Nordic region and elsewhere.

## Introduction

An estimated 71 million people worldwide have chronic hepatitis C virus (HCV) infection, including 14 million people in the World Health Organization (WHO) European Region. [1,2] In this region, HCV causes more than 100,000 deaths annually. [3] Nearly 3.3 million people in the countries of the European Union are living with chronic HCV infection. [4] People who inject drugs (PWID), including those who inject currently and those who have done so in the past, are the primary group affected by HCV in Europe. The incidence and prevalence of HCV infection remain high among PWID in most countries, while access to prevention and harm reduction services varies widely. [3,5–7] Approximately 43% of the 1.2 million PWID in the European Union/European Free Trade Association region have HCV RNA, which indicates active HCV infection. [8] PWID worldwide have high incarceration rates, and injection drug use is common in prison settings. [9,10] For these and other reasons, the HCV disease burden is likely to be large among prison populations worldwide. [11] Experts have called for European countries to prioritise the management of HCV infection among PWID through policies and guidelines specifically targeting this population. [3,5,12–16]

The five Nordic countries (Denmark, Finland, Iceland, Norway and Sweden) all have chronic HCV prevalence levels well below 1% in the general population, and the estimated numbers of chronic HCV cases in these countries collectively total only 100,000. [1] However, the disease burden is highly concentrated in PWID populations. In Denmark, for example, 68% to 75% of people living with chronic HCV infection in 2014 acquired the disease by sharing injecting drug equipment. [17] In Sweden, 65% of new cases of HCV infection reported to the authorities are attributable to the sharing of injecting drug equipment. [18] In Iceland, while the estimated prevalence of chronic HCV infection is 0.3%, 85% of cases are among people with a history of injecting drug use. [19] In 2015, Finland had 23,000 chronic HCV cases, a figure representing 0.5% of the country’s total population. [20] Approximately half of these cases are active injecting drug users. [21]

In 2016, WHO Member States adopted the goal of eliminating HCV as a major public health threat by 2030, defining this as an 80% reduction in new chronic infections and a 65% reduction in mortality. WHO also identified a series of “priority actions” for combating viral hepatitis and called on countries to implement the actions that speak to specific country-level priorities. [22] There is scarce published information on how the Nordic countries are addressing HCV at the policy level, apart from a 2016 description of Iceland’s national treatment-as-prevention campaign on the website clinicaltrials.gov. [23] Two central sources of information on national responses to HCV, the 2013 *Global policy report on the prevention and control of viral hepatitis in WHO Member States* [24] and the 2014 *Global community hepatitis policy report*, [25] contain limited information from the Nordic countries, and furthermore, it is not known whether this information is still current. Thus, it is not possible to ascertain the extent to which the Nordic countries are prepared to move toward HCV elimination.

We sought to examine the HCV policy landscape in Denmark, Finland, Iceland, Norway and Sweden in order to inform the ongoing discourse about how Nordic governments should address this disease. In recognition of previous disagreements among different types of stakeholders regarding national viral hepatitis policies, [26] the study design called for information to be gathered from representatives of governments, medical societies and civil society organisations (CSOs).

## Methods

We developed a cross-sectional, English-language survey through a two-step process in 2016. We conducted desk research to identify the most notable HCV policy issues facing national governments generally and the governments of the Nordic countries particularly. We then drafted survey questions to reflect these issues and shared them with the Hep-Nordic study group, which is comprised of either one or two viral hepatitis experts from each of the five study countries. Multiple rounds of input from Hep-Nordic study group members led to extensive revisions over a period of five months, until both the study group and the research team found the final survey questions to be suitable for capturing the relevant policy information. We piloted the study instrument to three individuals before the start of the study. Two of them lived in Hep-Nordic study countries and were knowledgeable about their countries’ responses to viral hepatitis.

In consultation with the Hep-Nordic study group, we used a purposive sampling process intended to identify four stakeholders per country representing four categories: ministries of health or similarly relevant government agencies, hepatitis patient groups, drug user groups and national medical societies. Formal sample size calculations were not appropriate for this study design due to its exploratory nature. Before beginning the enrolment process, we decided that Hep-Nordic study group members would be eligible to serve also as survey respondents, as we anticipated the potential difficulty of identifying an adequate number of suitable survey respondents, given the relatively small size of the stakeholder communities in some Nordic countries. Ultimately, two individuals served as both study group members and study participants. The sampling process identified a total of 17 stakeholders, three fewer than were sought. We were unable to make contact with anyone who could represent the following types of stakeholders: a hepatitis patient group in Iceland, a drug user group in Iceland, and a drug user group in Norway.

We created and managed the final survey (S1 Appendix) using the web-based electronic data collection tool Research Electronic Data Capture (REDCap), [27] hosted online by the Centre for Health and Infectious Disease Research (CHIP), Division of Infectious Diseases, Rigshospitalet, University of Copenhagen, Denmark. We invited the selected key stakeholders to complete the survey online using a customised survey link auto-generated by the REDCap system and distributed to each individual via email. Data were collected in October–November 2016.

The survey contained 21 HCV policy questions organised into four domains: national coordination (four items), prevention (four items), testing and linkage to care (six items) and treatment (seven items). Some questions also had sub-questions. All but one of the main questions were closed-ended questions, with most asking respondents to choose between the answers “Yes”, “No” and “Do not know.” Most sub-questions were also closed-ended, although respondents were given opportunities to add comments to clarify their answers. Respondents were requested to provide sources for their responses where possible.

Following survey completion, we exported the full dataset from REDCap to Microsoft Excel for data cleaning, resolution of queries and descriptive analyses. As part of data cleaning, we initially reviewed the full dataset to identify instances where different respondents from the same country had provided conflicting answers to survey questions. Discrepant responses were resolved by accepting the majority response and/or the response supported by a valid source. In cases in which there was no majority response, or where no sources were mentioned, or we could not determine the correct answer to the question, we consulted with the Hep-Nordic study group participant(s) from the relevant country. Given the frequency with which stakeholders contradicted each other in their survey responses, we decided to analyse the discrepancies for inclusion in the overall study findings.

All descriptive analyses were performed in Microsoft Excel 2016 (version 15.26). For analysis purposes, we combined hepatitis patient groups and drug user groups into “civil society organisations”. We analysed partial responses and “Do not know” responses as incorrect and counted these as discrepancies. We analysed responses by individual country, survey domain and stakeholder group. It was possible for respondents to be asked different numbers of sub-questions depending on which answers they provided to the main questions; therefore, calculations vary for each country. We excluded responses to one primary question and one sub-question from the final analysis due to a demonstrated lack of understanding of the intended meaning of those questions.

According to the regional representative of the Danish data protection agency and the Barcelona Institute for Global Health (ISGlobal), this study was not considered to be research on human subjects and therefore did not require ethical review or approval. We stored all data on secure servers in the Capital Region of Denmark and the data were managed according to Danish regulations.

## Results

Of the 17 stakeholders who were identified and invited to participate in the study, 14 agreed to participate, yielding an 82% response rate.

### National coordination

Respondents from three of the five countries (Finland, Iceland and Norway) reported that a written national strategy for viral hepatitis exists, approved by the national government (Table 1). One of the three countries (Iceland) reported the existence of an action plan for strategy implementation. Stakeholders from the two countries without national written strategies (Denmark and Sweden) reported that they do not have plans to develop strategies. While Finland and Iceland reported that their national strategies address only HCV, Norway reported having a strategy that addresses all forms of viral hepatitis. Both Finland and Norway reported that CSOs were consulted during the development of the strategy.

**Table 1:** NATIONAL COORDINATION.

	Denmark		Finland		Iceland		Norway		Sweden	
Question	Answer	Discrepancy	Answer	Discrepancy	Answer	Discrepancy	Answer	Discrepancy	Answer	Discrepancy
Written national viral hepatitis strategy approved by the national government	No^1^	Yes	Yes^2^	N/A	Yes^3^	N/A	Yes	No	No	No
exclusively for viral hepatitis	N/A	N/A	Yes	N/A	Yes	N/A	Yes	No	N/A	N/A
addresses hepatitis A virus	N/A	N/A	No	N/A	No	N/A	Yes	No	N/A	N/A
addresses hepatitis B virus	N/A	N/A	No	N/A	No	N/A	Yes	No	N/A	N/A
addresses hepatitis C virus	N/A	N/A	Yes	N/A	Yes	N/A	Yes	No	N/A	N/A
addresses hepatitis D virus	N/A	N/A	No	N/A	No	N/A	Yes	No	N/A	N/A
addresses hepatitis E virus	N/A	N/A	No	N/A	No	N/A	Yes	No	N/A	N/A
addresses raising public awareness	N/A	N/A	Yes	N/A	Yes	N/A	Yes	No	N/A	N/A
addresses surveillance	N/A	N/A	Yes	N/A	Yes	N/A	Yes	No	N/A	N/A
addresses vaccination	N/A	N/A	No	N/A	No	N/A	Yes	No	N/A	N/A
addresses prevention of transmission generally	N/A	N/A	Yes	N/A	Yes	N/A	Yes	No	N/A	N/A
addresses prevention of transmission via injecting drug use	N/A	N/A	Yes	N/A	Yes	N/A	Yes	No	N/A	N/A
addresses prevention of transmission in health care settings	N/A	N/A	No	N/A	No	N/A	Yes	Yes	N/A	N/A
addresses diagnostic testing	N/A	N/A	Yes	N/A	Yes	N/A	Yes	Yes	N/A	N/A
addresses linkage to care for people diagnosed with viral hepatitis	N/A	N/A	Yes	N/A	Yes	N/A	Yes	No	N/A	N/A
addresses treatment and care for people diagnosed with viral hepatitis	N/A	N/A	Yes	N/A	Yes	N/A	Yes	No	N/A	N/A
addresses HIV co-infection	N/A	N/A	No	N/A	Yes	N/A	No	No	N/A	N/A
developed in consultation with civil society groups	N/A	N/A	Yes	N/A	No	N/A	Yes	No	N/A	N/A
Action plan on how the strategy will be implemented	N/A	N/A	No	N/A	Yes	N/A	No	No	N/A	N/A
Development of national viral hepatitis strategy is planned	No	Yes	N/A	N/A	N/A	N/A	N/A	N/A	No	Yes
National goal for the elimination of HCV	No	No	No	Yes	Yes	N/A	No	No	No	No
National disease register for HCV infection	No	Yes	No	Yes	Yes	No	Yes	Yes	Yes	Yes
Government employs geographic information system (GIS) for disease monitoring	No	Yes	Yes, but does not include viral hepatitis data	Yes	No	No	Yes, but does not include viral hepatitis data	No	Yes, includes viral hepatitis data	Yes

1. There are guidelines from the Danish Societies of Infectious Diseases and Gastroenterology & Hepatology, but there is no written national strategy from the National Board of Health.

2. Strategy was released at the time of the survey. Answers in the table reflect information captured from a survey respondent with knowledge of the strategy.

3. Respondents provided examples of two different national strategies (one from the government and one from the TraPHepC project). For the purpose of this analysis, the answers captured reflect the TraPHepC project strategy and therefore, discrepancies were not observed.

Survey respondents from countries with strategies were further asked if those strategies addressed several specific elements of the recommended public health response to viral hepatitis, including public awareness, surveillance, vaccination, transmission prevention, diagnostic testing, linkage to care, treatment, and HIV co-infection. Respondents’ answers to these questions suggest that Norway’s strategy is the most comprehensive, as it was reported to encompass all elements except for HIV co-infection. Only the Iceland strategy was reported to address HIV co-infection.

The only study country reported to have a national goal regarding the elimination of HCV was Iceland.

Stakeholders from Iceland, Norway and Sweden reported that their governments, or government-related institutions, have national disease registries for HCV.

Stakeholders from Finland, Norway and Sweden responded affirmatively when asked “*Does your government employ a geographic information system (GIS) in its disease monitoring activities*?*”*, although only the Swedish stakeholders indicated that viral hepatitis data are included in analyses.

### Hepatitis C prevention

A survey question asked if the government or any government-related institution had conducted or funded another organisation to conduct public awareness/education campaigns relating specifically to HCV prevention since January 2015. All countries with the exception of Denmark answered “yes” (Table 2).

**Table 2:** HEPATITIS C PREVENTION.

	Denmark		Finland		Iceland		Norway		Sweden	
Question	Answer	Discrepancy	Answer	Discrepancy	Answer	Discrepancy	Answer	Discrepancy	Answer	Discrepancy
Public awareness/education campaigns held specifically on HCV prevention (since January 2015)	No	Yes	Yes	Yes	Yes	No	Yes	No	Yes	Yes
Opioid substitution therapy available to the general public in all parts of the country	Yes	No	Yes	No	Yes	Yes	Yes	No	Yes	No
Needle and syringe exchange programmes available to the general public in all parts of the country	Yes	Yes	Yes	Yes	No–only in some parts	No	Yes	No	No–only in some parts	No
General public can participate anonymously in needle and syringe exchange programmes	Yes	No	Yes	No	Yes	No	Yes	No	No–Registration is required	Yes
Minimum age for participating in needle and syringe exchange programmes	No	No	No	Yes	No	No	No	No	Yes	No
Age at which general public can participate in needle and syringe exchange programmes	N/A	N/A	N/A	Yes	N/A	N/A	N/A	N/A	18^1^	No
Opioid substitution therapy programmes provided in prison facilities in all parts of the country	Yes	No	No–only in some parts	Yes	Yes	Yes	No–only in some parts	Yes	No–only in some parts	Yes
Needle and syringe exchange programmes provided in prison facilities in all parts of the country	No–not anywhere	Yes	No–not anywhere	Yes	No–not anywhere	No	No–not anywhere	Yes	No–not anywhere	Yes
Bleach and other materials for sterilising injecting equipment provided in prison facilities in all parts of the country	Yes	Yes	No–not anywhere	Yes	No–not anywhere	No	No–only in some parts	Yes	No–not anywhere	Yes
Clean needles and syringes legally available to people who inject drugs outside of needle and exchange programmes (e.g. at pharmacies)	Yes	Yes	Yes	Yes	Yes	No	Yes	Yes	No	Yes

1. Policy recently updated, minimum age lowered from 20 to 18 years.

All five countries reported that opioid substitution therapy (OST) is available to the general public in all parts of their countries. Respondents from Denmark, Finland and Norway indicated that needle and syringe programmes (NSPs) are available to the general public in all parts of their countries. Respondents from Iceland and Sweden indicated that these services are available only in some parts of their countries. It was reported that people may participate in NSPs anonymously in all countries except Sweden, where registration is required. Sweden was reported to be the only country with a minimum age requirement for participating in NSPs.

Denmark and Iceland reported that OST is provided in prison facilities in all parts of their countries. Finland, Norway and Sweden reported that OST is provided in prison facilities in some parts of their countries. None of the respondents reported the provision of NSPs in prison facilities. Only Denmark reported that bleach and other materials for sterilising injecting equipment are available in prison facilities in all parts of their countries. Norway reported that while the materials may be provided in some facilities, they are not available in all parts of the country.

Respondents in every country except Sweden reported that clean needles and syringes can be legally obtained outside of NSP programmes.

### Hepatitis C testing and linkage to care

**Fig 1:**
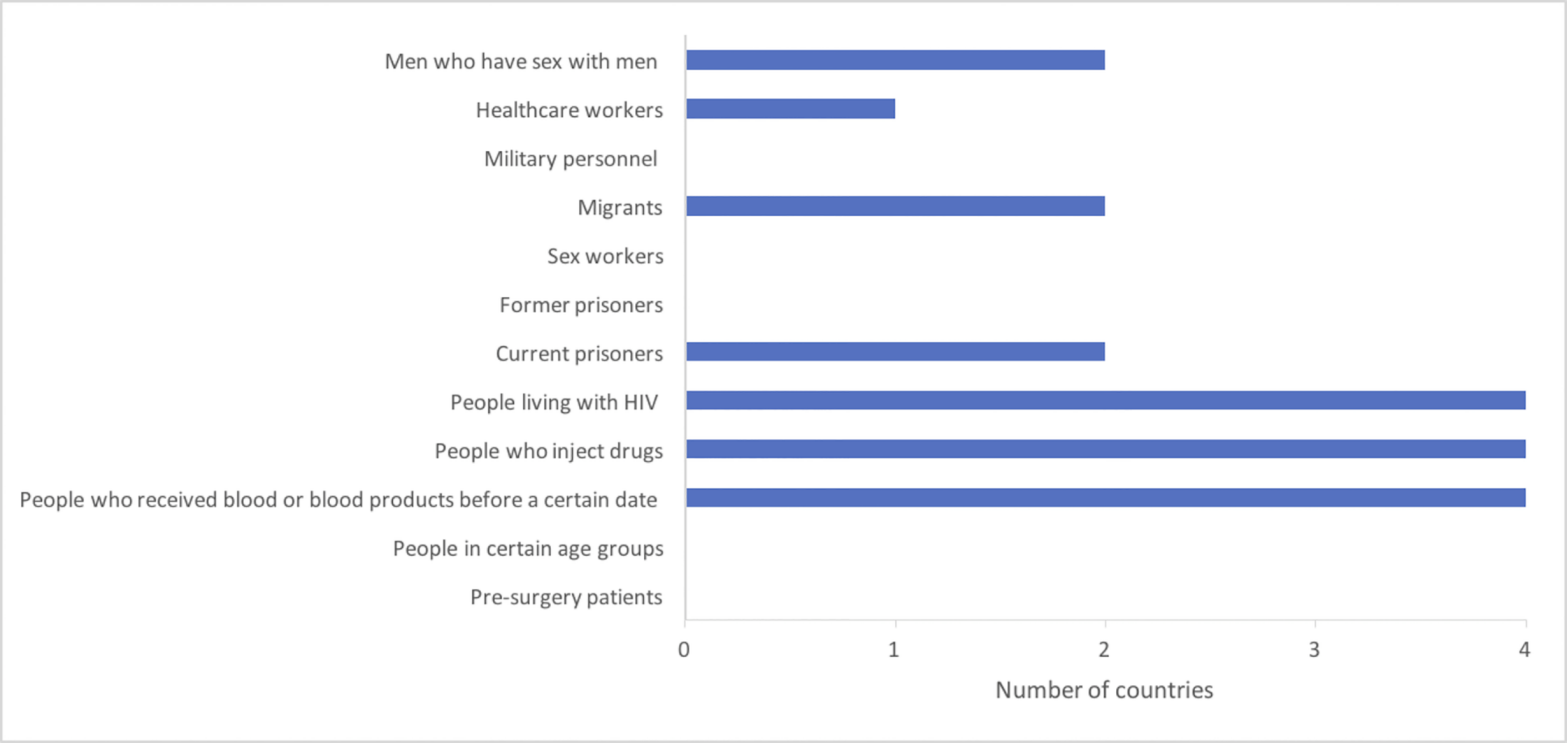
Groups that should be routinely offered HCV testing according to national guidelines.

Stakeholders from four countries reported that national guidelines identified certain groups whose members should be routinely offered HCV testing, with Finland being the only country without such guidelines (Table 3). All four countries with national guidelines recommend that people who received blood or blood products before a certain date, PWID, and people living with HIV should be routinely offered HCV testing (Fig 1). In two countries (Iceland and Norway), national guidelines identify current prisoners, migrants and men who have sex with men as groups that should be routinely offered HCV testing. Only one country’s guidelines (Denmark) make this recommendation for health care workers. No country’s guidelines call for HCV testing to be routinely offered to people in certain age groups, or to pre-surgery patients, sex workers, former prisoners, or military personnel.

**Table 3:** HEPATITIS C TESTING AND LINKAGE TO CARE.

	Denmark		Finland		Iceland		Norway		Sweden	
Question	Answer	Discrepancy	Answer	Discrepancy	Answer	Discrepancy	Answer	Discrepancy	Answer	Discrepancy
Which groups are identified in the national guidelines as groups that should routinely be offered HCV testing?										
pre-surgery patients	No	No	N/A^1^	N/A	No	No	No	No	No	No
all people in certain age groups	No	No	N/A	N/A	No	No	No	No	No	No
people who received blood or blood products before a certain date	Yes	Yes	N/A	N/A	Yes	No	Yes	No	Yes	No
people who inject drugs	Yes	No	N/A	N/A	Yes	No	Yes	No	Yes	Yes
people living with HIV	Yes	Yes	N/A	N/A	Yes	No	Yes	No	Yes	Yes
people who are currently incarcerated	No	Yes	N/A	N/A	Yes	Yes	Yes	No	No	Yes
former prisoners	No	Yes	N/A	N/A	No	No	No	No	No	No
sex workers	No	Yes	N/A	N/A	No	No	No	No	No	No
migrants	No	Yes	N/A	N/A	Yes	Yes	Yes	No	No	Yes
military personnel	No	No	N/A	N/A	No	No	No	No	No	No
healthcare workers	Yes	Yes	N/A	N/A	No	No	No	No	No	No
men who have sex with men	No	Yes	N/A	N/A	Yes	Yes	Yes	No	No	Yes
People in all parts of the country have access to free HCV testing services	Yes	Yes	Yes	Yes	DNK	Yes	Yes	No	Yes	No
People in all parts of the country have access to anonymous HCV testing services	No	No	No	Yes	No	No	No	Yes	No	Yes
Rapid HCV testing is available in community settings	No	Yes	No	Yes	Yes	Yes	No	No	No	Yes
There is a clear linkage-to-care mechanism	Yes	No	No	Yes	Yes	No	Yes	No	Yes	Yes

1. Respondent indicated that there are no official national HCV testing guidelines in Finland. Therefore, these answers are not currently reflected in the table.

Stakeholders from every country except Iceland indicated that people in all parts of their countries have access to free HCV testing. Respondents from all five countries reported that access to anonymous HCV testing services is not available in all parts of their countries. Respondents from Iceland were the only ones to report that rapid HCV testing is available in community settings.

Respondents from every country except Finland reported the existence of a clear linkage-to-care mechanism to ensure that people who are diagnosed with HCV are referred directly to a physician who can manage their care.

### Hepatitis C treatment

Finnish stakeholders were the only ones who reported not having national HCV treatment guidelines (Table 4). Participants representing all five of the study countries reported the provision of publicly funded direct-acting antiviral (DAA) treatment for patients with chronic HCV infection. According to stakeholders from Iceland, there has been unrestricted access to DAA treatment since 2016. Other study countries limit access to DAA treatment, for instance, to those who have a minimum fibrosis level or by setting patient quotas.

**Table 4:** HEPATITIS C TREATMENT.

	Denmark		Finland		Iceland		Norway		Sweden	
Question	Answer	Discrepancy	Answer	Discrepancy	Answer	Discrepancy	Answer	Discrepancy	Answer	Discrepancy
National guidelines for the treatment of HCV	Yes	Yes	No^1^	Yes	Yes	No	Yes	No	Yes	No
Guidelines published by European Association for the Study of the Liver (EASL) or other international clinical association are adopted as national guidelines	No	No	N/A	N/A	No	No	No	No	No	No
Guidelines published by World Health Organization (WHO) are adopted as national guidelines	No	No	N/A	N/A	No	No	No	No	No	No
National government develops its own national guidelines	Yes	Yes	N/A	N/A	Yes^2^	Yes	No	No	No	Yes
National medical society develops its own national guidelines	Yes	Yes	N/A	N/A	No	No	Yes	No	Yes	Yes
Other publishers of guidelines	N/A	N/A	N/A	N/A	TraPHepC project	Yes	N/A	N/A	N/A	N/A
Most common HCV genotype in country	Genotype 1	Yes	Genotype 3	Yes	Genotype 3	No	Genotype 3	No	Genotype 1	Yes
Duration of recommended first-line treatment regimen for this genotype	12 weeks	Yes	12 weeks	Yes	12 weeks	No	Other^3^	No	12 weeks	Yes
Second most common HCV genotype in country	Genotype 3	Yes	Genotype 2	Yes	Genotype 1	No	Genotype 1	No	Genotype 3	Yes
Duration of recommended first-line treatment regimen for this genotype	12 weeks	Yes	-	Yes	12 weeks	No	12 weeks	Yes	12 weeks	Yes
Publicly funded direct-acting antiviral (DAA) treatment provided to chronic HCV patients in your country	Yes	No	Yes	No	Yes	No	Yes	No	Yes	Yes
Only patients above a certain fibrosis level are eligible for treatment	Yes	No	Yes	No	No	No	Yes^4^	No	Yes	Yes
Only a limited number of patients can be treated within a certain time period or a certain geographical area	Yes	Yes	Yes	No	No	No	No	No	No	No
People who currently drink alcohol are not treated	No	Yes	Yes	Yes	No	No	No	No	No	No
People who injected drugs in the past are not treated, even if they are not currently injecting drugs	No	No	No	No	No	No	No	No	No	No
People who currently inject drugs are not treated	Yes	Yes	Yes	Yes	No	No	No	No	No	Yes
People who injected drugs in the past are only treated if they have abstained from injecting drugs for a specified period of time	No	Yes	Yes	Yes	No	No	No	No	No	Yes
People who currently inject drugs or injected drugs in the past are treated only if they are receiving opioid substitution therapy	Yes	No	Yes	Yes	No	No	No	No	No	No
HCV patients have the option of being treated in non-hospital settings	Yes	Yes	Yes	Yes	Yes	Yes	Yes	No	No	Yes
HCV patients have the option of being treated in general practitioner clinics	No	No	No	No	No	No	No	No	No	No
HCV patients have the option of being treated in addiction/opioid substitution clinics or harm reduction centres	Yes	Yes	No	No	Yes	Yes	Yes	No	No	No
HCV patients have the option of being treated in “other” settings	Yes	Yes	Yes	Yes	Yes	No	No	No	Yes	Yes
“Other” settings in which HCV patients have the option of being treated	Prisons, in collaboration with the hospital	No	Private practice gastroenterologists, self-paid by patients	Yes	Prisons	Yes	N/A	N/A	Prisons	Yes
HCV treatment can be obtained from healthcare providers in all parts of the country	Yes	Yes	Yes	Yes	No	Yes	Yes	No	Yes	Yes
HCV treatment is provided in prisons in all parts of the country	No	Yes	No	No	No	Yes	No	Yes	No	Yes

1. There are currently no official national guidelines for the treatment of HCV. Treatment recommendations made by leading gastroenterologists are published in the Finnish medical journal. Establishing national guidelines, including treatment, are priorities for the new hepatitis C strategy published in November 2016.

2. Guidelines from the national government and from the TraPHepC Project were both reported in the survey.

3. Treatment is offered only to patients below 40 years of age. If HCV RNA is not positive after 4 weeks, the treatment is extended to 12 weeks

4. Yes” for genotypes 2 and 3 and “No” for genotype 1.

In all countries except Sweden, patients with chronic HCV infection have the option of being treated in various non-hospital settings. None of the study countries give patients the option of being treated by general practitioners, though in Denmark, Iceland and Norway, patients have the option of being treated in addiction/opioid substitution clinics or harm reduction centres. Danish, Icelandic and Swedish respondents reported that HCV patients in their countries have the option of being treated in prison.

Stakeholders from four countries indicated that HCV treatment can be obtained from healthcare providers in all parts of their countries. While that is not the case in Iceland, patients there are eligible to receive free transportation to Reykjavik for care if needed. At the time of the survey, none of the countries reported the availability of HCV treatment in prison facilities in all parts of their countries.

### Discrepancies between responses from representatives of different stakeholder groups

**Fig 2:**
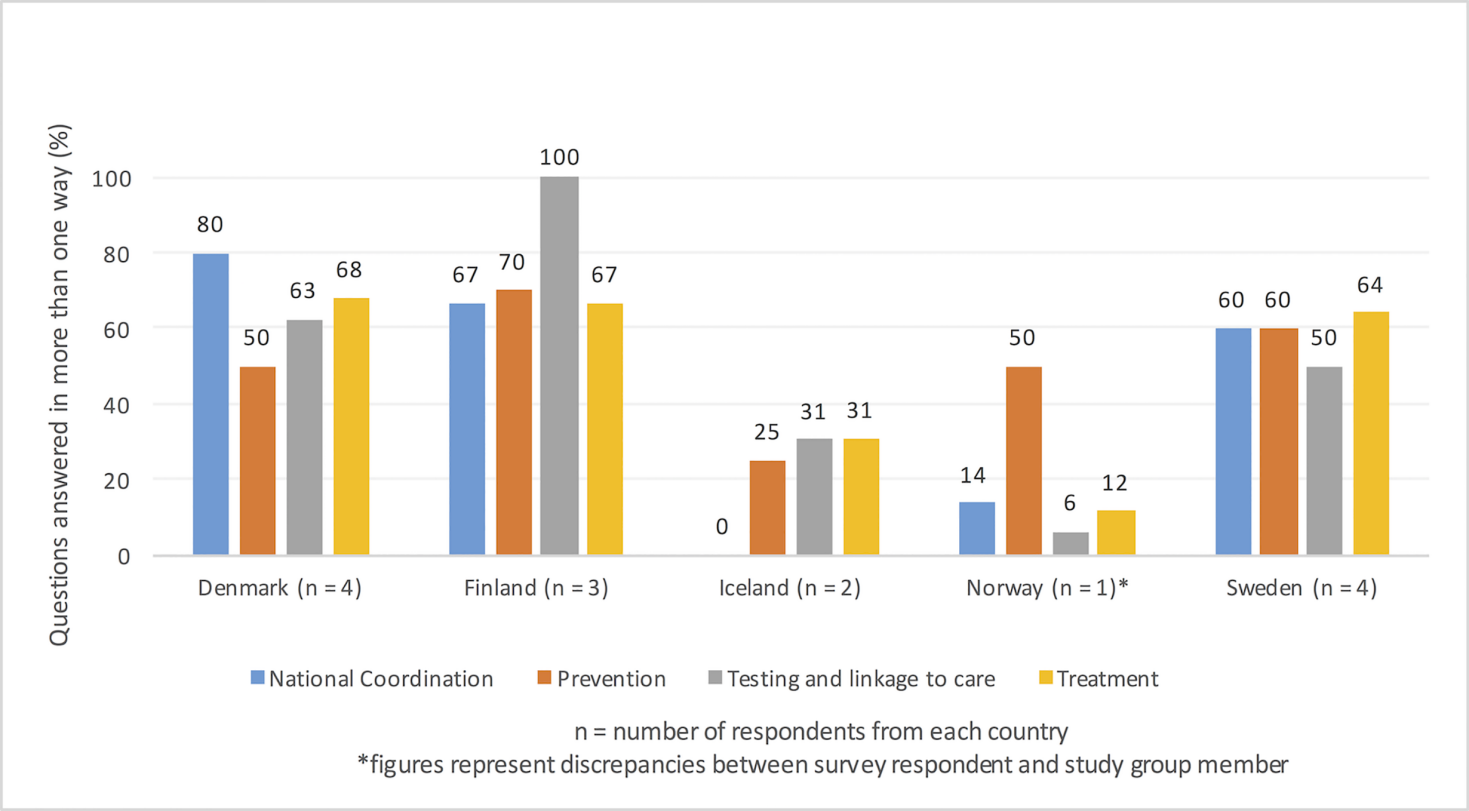
Incidence of stakeholder disagreement by country and survey domain.

The incidence of stakeholder disagreement by country and survey domain ranged from 0 for the “national coordination” domain in Iceland to 100% for the “testing and linkage to care” domain in Finland, with 50% or more disagreement for the majority of domains across the countries (Fig 2). None of the individual stakeholders from any of the countries gave correct answers to all of the survey questions. On average, representatives of CSOs had a higher proportion of incorrect responses to survey questions (39%) than representatives of government or medical societies (data not shown).

## Discussion

This study sought to provide insight into the policy response to HCV in the Nordic countries, where transmission among PWID contributes greatly to the HCV disease burden. Although these are among the wealthiest countries in the world, and are well recognised for their responsive health systems, our study suggests that the Nordic region as a whole has not consistently expressed its full commitment to tackling the HCV epidemic at the policy level. There are gaps in relation to the existence of national viral hepatitis strategies and national HCV treatment guidelines, as well as uneven efforts to address HCV prevention, testing and linkage to care. The provision of publicly funded DAA treatment puts all five countries in a good position to pursue global HCV elimination targets; yet other policy shortcomings have the potential to undermine progress in all five countries. Furthermore, not all key stakeholders within the study countries have the same information regarding which policies are actually in place.

With the inclusion of a goal addressing viral hepatitis in the Sustainable Development Goals in 2016, the United Nations signalled that governments need to collectively step up the global response to this group of diseases. [28] Later the same year, WHO made the charge to governments more concrete by introducing the first *Global health sector strategy on viral hepatitis*. [22] The *Action plan for the health sector response to viral hepatitis in the WHO European Region*, which is aligned with the Global Strategy, adds targets for European countries, such as “50% of people living with chronic HBV and HCV infections are diagnosed and aware of their condition”. [3] Governments must implement multiple types of public health interventions on a national scale in order to progress towards HCV elimination, as reflected in the “continuum of viral hepatitis services” described in the Global Strategy. National policies can greatly influence the extent to which the necessary interventions are brought to scale in all areas of the HCV continuum, including prevention, testing, linkage to care and treatment, as well as prevention of reinfection among people who are cured, which the *Action Plan* has established as a priority for all European countries.

In light of their abundant resources and strong public health infrastructure, [29] the Nordic countries are in a position to demonstrate global leadership on HCV elimination. High levels of HCV transmission among PWID in the Nordic region provide both a moral and practical imperative for governments to act decisively, and the effectiveness of new DAA treatment regimens makes the full elimination of HCV much more feasible than it was even five years ago. Our study findings highlight policy gaps across the HCV continuum and suggest that all of the Nordic countries should consider strengthening their policy responses to HCV in one or more ways. With neither written national strategies nor plans to develop such strategies, the governments of Denmark and Sweden in particular demonstrate a lack of commitment to HCV elimination.

One unexpected finding from our study was the widespread lack of consensus among respondents about their countries’ policy responses to HCV. This raises the question of whether there is sufficient communication among stakeholders regarding the formulation or implementation of policies. CSOs appeared to be lacking more information than other stakeholder groups, which is problematic, as WHO recognises the involvement of affected communities as an important component of strong national responses to viral hepatitis. [22]

Current best practices call for PWID to have access to comprehensive, evidence-based multidisciplinary harm reduction services, especially OST and sterile injecting equipment, along with community-based support services. [30] The *Action plan for the health sector response to viral hepatitis in the WHO European Region* recognises the centrality of harm reduction as a component of the Region’s viral hepatitis elimination efforts by calling for the following milestone to be reached by 2018: “Policies developed and implemented supporting comprehensive harm reduction programmes, including risk reduction communication, needle and syringe programmes, and opioid substitution therapy or ‘pharmacotherapy of opioid dependence’ including in the community and in prisons”. [3] Our finding that the availability of harm reduction services varies widely across the Nordic countries is thus notable. While all countries reported the availability of OST to the general public, other key services are limited, as are services in prison facilities. For example, Sweden–the Nordic country with the largest number of patients receiving HCV treatment–has several policies that limit access to harm reduction services. It is the only study country requiring a minimum age to access NSPs, the only one where clean needles/syringes cannot be purchased legally at pharmacies by PWID, and the only one that does not allow anonymous participation in NSPs. In contrast, a respondent from Finland reported that needles and syringes can be purchased without a medical prescription at most pharmacies in the country, and that “pharmacies play a key role in needle and syringe provision in areas [without] health counselling centres”.

The 2016 guidelines from the European Association for the Study of the Liver (EASL) state that all patients with HCV infection “must be considered for treatment”, [16] and in 2017, EASL publicly noted that this recommendation is intended to apply to PWID alongside other patients. [31] Our study findings confirm that most of the Nordic countries are at the forefront of HCV treatment efforts through the use of DAAs to attain high cure rates in those with chronic HCV infection, including PWID, as all countries provide publicly funded DAA treatment. For example, in 2016, Iceland initiated a nationwide campaign whereby all viraemic patients were offered DAA treatment free of charge and without restrictions, and the country launched an intensive HCV testing and treatment effort in prisons. In other Nordic countries, however, treatment availability for prison populations is inconsistent, which is problematic, in light of what is known about the HCV burden in prisons worldwide. [11]

National HCV programmes must have reliable epidemiological and service coverage information in order to determine which interventions need to be intensified in which populations and locations. [22] Our study revealed potential opportunities for harmonisation between data from geographic information systems and national disease registries, and for improved disease surveillance and service delivery monitoring.

### Limitations

Our analysis may be limited by the survey sample, as not all stakeholder groups were evenly represented. The small sample size precludes viewing this study as anything more definitive than an exploratory effort to inform stakeholders’ discussions about the public health responses to HCV in the five study countries. It is possible that the use of English for the survey may have resulted in a lack of clarity or in questions being interpreted in different ways by different respondents; in fact, two questions were ultimately eliminated from the analysis because of confusion among respondents about their meaning. Some of the questions that were asked may have been more difficult to answer in an informed way for respondents outside of government than for government respondents. Although all survey respondents were told that what they shared would be reported anonymously, the small size of the national stakeholder community in all five study countries raises the question of whether some respondents may not have perceived themselves to be truly anonymous. It is possible that concerns about the perceptions of other stakeholders may have influenced their answers to survey questions. In addition, this analysis may not reflect policy changes that occurred after the study participants submitted their survey responses and indeed Finland has since approved a viral hepatitis strategy.

## Conclusions

Our study, the first to investigate the policy response to HCV in all of the Nordic countries, has highlighted areas in which policy-makers in these countries should consider strengthening their responses. In all countries, we observed widespread disagreement between key stakeholders regarding which policies are in place. The study findings may inform efforts in other countries where PWID are disproportionately affected by HCV. Stakeholder alignment and an established elimination goal with an accompanying strategy and implementation plan should be recognised as the basis for coordinated national public health efforts to achieve HCV elimination in the Nordic region and elsewhere.

## Supporting information

S1 Appendix Hep-Nordic survey. (PDF)
