# Supplementary material for: Policy responses to hepatitis C in the Nordic countries: Gaps and discrepant reporting in the Hep-Nordic study
Source: PLoS One. 2018 Jan 30;13(1):e0190146. doi: 10.1371/journal.pone.0190146 (PMC5790214; doi:10.1371/journal.pone.0190146)
Supplement: S1 Appendix — (PDF) [file pone.0190146.s001.pdf]

Hep-Nordic: Assessing the Policy Response to Hepatitis C in the Nordic Countries

Thank you for participating in the 2016 Hep-Nordic hepatitis C policy survey, which is being conducted by a research team based at the WHO Collaborating Centre on HIV and Viral Hepatitis, Rigshospitalet, the University of Copenhagen, together with an international study group.

Please follow these guidelines for completing the survey:

To move from one section to another, please use the arrow buttons at the end of each section. Please do not use the “back” and “forward” buttons in your browser.

Please fully answer all questions in English.

Please provide only one answer per question, unless otherwise instructed.

If you do not know the answer to a question, please make every effort to find the information.

When possible, please indicate where you got your information in the “sources” field at the end of each question. Sources might include – but are not limited to – government website pages, published reports, and e-mail communication with national experts.

You may save your work on an incomplete survey and return to it at another date. To do this, use the “Save & Return Later” button that appears on all pages of the survey.

Please ensure that you have fully answered all questions before clicking on the “Submit” button that appears on the last page of the survey.

A PDF of the survey is available for reference only. The actual survey must be completed online. To download the PDF, use the link below.

If you have any questions or concerns about the Hep-Nordic study, please contact Principal Investigator Jeffrey V. Lazarus of the University of Copenhagen at jeffrey.lazarus@regionh.dk.

@HIDDEN TODAY __________________________________

This PDF of the survey is available for reference only. The survey must be completed online.

[Attachment: "HepNordic_2016_Survey_Instrument.pdf"]

SECTION 1. CONTACT INFORMATION

All contact information will remain private, and all responses to the survey will be reported anonymously.

First name: ________________________________

Last name: ________________________________

Type of organisation:

Please choose the one answer that best describes your organisation.

- ◯ Government agency, national level
- ◯ Government agency, subnational level
- ◯ NGO: hepatitis patient group
- ◯ NGO: drug user group
- ◯ NGO: direct service provider
- ◯ NGO: other (please specify below)
- ◯ Medical society
- ◯ University-based research programme
- ◯ Hospital or medical clinic
- ◯ Private foundation
- ◯ Other (please specify below)

Other NGO -- please specify: ________________________________

Other -- please specify: ________________________________

Name of organisation: ________________________________

Organisational role/title/position of person completing survey: ________________________________

Street address: ________________________________

City: ________________________________

Postal code: ________________________________

Country: ________________________________

Phone number: ________________________________

E-mail address: ________________________________

SECTION 2. NATIONAL COORDINATION

2.1 Written national viral hepatitis strategy

Does your country have a written national viral hepatitis strategy that has been approved by the national government?

◯ Yes
◯ No
◯ Do not know

Is the strategy exclusively for viral hepatitis, or does it integrate viral hepatitis with other diseases?

◯ It is exclusively for viral hepatitis
◯ It integrates viral hepatitis with other diseases
◯ Do not know

Which forms of viral hepatitis does the strategy address?

Please choose all answers that apply.

☐ Hepatitis A virus
☐ Hepatitis B virus
☐ Hepatitis C virus
☐ Hepatitis D virus
☐ Hepatitis E virus

Which of the following topics are addressed in the strategy?

Please choose all answers that apply.

☐ Raising public awareness
☐ Surveillance
☐ Vaccination
☐ Prevention of transmission generally
☐ Prevention of transmission via injecting drug use
☐ Prevention of transmission in healthcare settings
☐ Diagnostic testing
☐ Linkage to care for people diagnosed with viral hepatitis
☐ Treatment and care
☐ HIV coinfection

Were civil society groups consulted during the development of the strategy?

(Civil society groups include non-governmental organisations and other groups that work independently of government to bring about improvements in a society such as improvements relating to healthcare.)

◯ Yes
◯ No
◯ Do not know

Is there an action plan stating how the strategy will be implemented?

◯ Yes
◯ No
◯ Do not know

Is planning underway to develop a national viral hepatitis strategy?

◯ Yes
◯ No
◯ Do not know

In your opinion, what are the main obstacles to establishing a national viral hepatitis strategy?

Please describe: ________________________________

In your opinion, what are the next steps that need to be taken to establish a national viral hepatitis strategy?

Please describe: ________________________________

Additional comments: ________________________________

Sources for answers: ____________________________

2.2 National goal regarding elimination of HCV

Has your country established a national goal regarding the elimination of HCV?

◯ Yes
◯ No
◯ Do not know

If yes, what is the goal? __________________________________

Additional comments:

____

Sources for answers: __________________________________

2.3 National disease register for HCV infection

Does your government or any government-related institution have a national disease register for HCV infection?

◯ Yes
◯ No
◯ Do not know

Additional comments:

____

Sources for answers: __________________________________

2.4 Government GIS in disease monitoring

Does your government employ a geographic information system (GIS) in its disease monitoring activities?

◯ Yes, has a GIS and includes viral hepatitis data in analyses
◯ Yes, has a GIS but does not routinely analyse viral hepatitis data
◯ No, does not have a GIS
◯ Do not know

Additional comments:

____

Sources for answers: __________________________________

SECTION 3. HEPATITIS C PREVENTION

3.1 Public awareness/education related to HCV prevention

Since January 2015, has your government or any government-related institution conducted or funded another organisation to conduct any public awareness/education campaigns relating specifically to HCV prevention?

◯ Yes
◯ No
◯ Do not know

Additional comments: ________________________________

Sources for answers: __________________________

3.2 Harm reduction services for the general public

Are the following services provided to the general public in all parts of your country?

	Yes	No - only in some parts of the country	No - not anywhere in the country	Do not know
Needle and syringe exchange programmes	◯	◯	◯	◯
Opioid substitution therapy	◯	◯	◯	◯

Can people participate anonymously in needle and syringe exchange programmes in your country? Or are they required to register by name?

◯ Participation is anonymous
◯ Registration is required
◯ Do not know

Is there a minimum age requirement for people to participate in needle and syringe exchange programmes in your country?

◯ Yes
◯ No
◯ Do not know

If yes, how old do people need to be to participate in needle and syringe exchange programmes?

◯ 15
◯ 16
◯ 17
◯ 18
◯ 19
◯ 20
◯ 21
◯ 22
◯ 23
◯ 24
◯ 25 or older

Additional comments: ________________________________

Sources for answers: __________________________

3.3 Harm reduction services in prisons

Are the following services provided in prison facilities in all parts of your country?

	Yes	No - only in some parts of the country	No - not anywhere in the country	Do not know
Needle and syringe exchange programmes	◯	◯	◯	◯
Opioid substitution therapy	◯	◯	◯	◯
Bleach and other materials for sterilising injecting equipment	◯	◯	◯	◯

Additional comments: ______________________________

Sources for answers: ______________________________

3.4 Harm reduction services outside of NSPs

Are clean needles and syringes legally available to people who inject drugs outside of needle and exchange programmes in your country? (e.g., at pharmacies)

◯ Yes
◯ No
◯ Do not know

Additional comments: ______________________________

Sources for answers: ______________________________

SECTION 4. HEPATITIS C TESTING & LINKAGE TO CARE

4.1 National guidelines for routine HCV testing

In your country, are any of the following groups identified in national guidelines as groups that should be routinely offered HCV testing?

Please choose all answers that apply.

- ☐ Pre-surgery patients
- ☐ All people in certain age group (please specify age group below)
- ☐ All people who received blood or blood products before a certain date (please specify date below)
- ☐ People who inject drugs
- ☐ People living with HIV
- ☐ Current prisoners
- ☐ Former prisoners
- ☐ Sex workers
- ☐ Migrants
- ☐ Military personnel
- ☐ Healthcare workers
- ☐ Men who have sex with men
- ☐ Other groups (please specify below)
- ☐ Unknown

Guidelines call for this age group to be routinely offered HCV testing: ________________________________

Guidelines call for all people who received blood or blood products before this date to be routinely offered HCV testing: ________________________________

Other groups identified in national guidelines as groups that should be routinely offered HCV testing: ________________________________

Additional comments:

____

Sources for answers: ________________________________

4.2 Low-barrier testing for high-risk populations

Are there any targeted low-barrier testing activities for high-risk populations in your country?

- ◯ Yes
- ◯ No
- ◯ Do not know

(In relation to HBV and HCV, high-risk populations are populations at higher risk than the general population of acquiring these diseases. Globally, HBV and HCV high-risk populations include people who inject drugs, men who have sex with men, transgender persons and sex workers. Additional high-risk populations such as migrants and prisoners may be identified in some settings depending on national and local HBV and HCV epidemic dynamics.)

Please give an example of an activity (1): ________________________________

Please give an example of an activity (2): ________________________________

Please give an example of an activity (3): ________________________________

Additional comments:

____

Sources for answers: ______________________________

4.3 Access to free HCV testing

Do people in all parts of your country have access to free HCV testing services?

- ◯ Yes
- ◯ No
- ◯ Do not know

Additional comments: ______________________________________

Sources for answers: ______________________________

4.4 Access to anonymous HCV testing

Do people in all parts of your country have access to anonymous HCV testing services?

- ◯ Yes
- ◯ No
- ◯ Do not know

Additional comments: ______________________________________

Sources for answers: ______________________________

4.5 Rapid HCV testing

Is rapid HCV testing available in community settings in your country?

- ◯ Yes
- ◯ No
- ◯ Do not know

Additional comments: ______________________________________

Sources for answers: ______________________________

4.6 Clear linkage-to-care mechanism

In your country, is there a clear linkage-to-care mechanism so that people who are diagnosed with HCV are referred directly to a physician who can manage their care? (Either a general practitioner/primary care physician or an appropriate specialist depending on your country's standard practice.)

(A linkage-to-care mechanism is a clearly defined process for helping people engage in medical care for HBV and HCV after they have been diagnosed with these diseases.)

- ◯ Yes
- ◯ No
- ◯ Do not know

Additional comments: ______________________________________

Sources for answers: ______________________________

SECTION 5. HEPATITIS C TREATMENT

5.1 Guidelines for HCV treatment

Does your country have national guidelines for the treatment of HCV?

- ◯ Yes
- ◯ No
- ◯ Do not know

Who publishes the guidelines?

Please choose all answers that apply.

- ☐ Guidelines by European Association for the Study of the Liver (EASL) or other international clinical association are adopted as national guidelines
- ☐ Guidelines by World Health Organization (WHO) are adopted as national guidelines
- ☐ National government develops its own national guidelines
- ☐ National medical society develops its own national guidelines
- ☐ Other (please specify below)
- ☐ Do not know

Other publishers of guidelines, please specify: ________________________________

Please indicate what the guidelines recommend as first-line treatment regimens for non-cirrhotic patients with the two most common HCV genotypes in your country.

Most common HCV genotype in your country

Most common HCV genotype in your country:

- ◯ Genotype 1
- ◯ Genotype 3
- ◯ Other genotype (please specify below)
- ◯ Do not know

Other genotype, please specify: ________________________________

Recommended first-line treatment regimen for non-cirrhotic patients with this genotype:

Drugs in regimen (1): ________________________________
(Please list one drug per field.)

Drugs in regimen (2): ________________________________
(Please list one drug per field.)

Drugs in regimen (3): ________________________________
(Please list one drug per field.)

Drugs in regimen (4): ________________________________
(Please list one drug per field.)

Duration of regimen:

- ◯ 8 weeks
- ◯ 12 weeks
- ◯ 24 weeks
- ◯ Other (please specify below)

Other duration of treatment regimen for non-cirrhotic patients with this genotype: ________________________________

Second most common HCV genotype in your country

Second most common HCV genotype in your country:

- ◯ Genotype 1
- ◯ Genotype 3
- ◯ Other genotype (please specify below)
- ◯ Do not know

Other genotype, please specify: __________________________________

Recommended first-line treatment regimen for non-cirrhotic patients with this genotype:

Drugs in regimen (1): __________________________________
(Please list one drug per field.)

Drugs in regimen (2): __________________________________
(Please list one drug per field.)

Drugs in regimen (3): __________________________________
(Please list one drug per field.)

Drugs in regimen (4): __________________________________
(Please list one drug per field.)

Duration of regimen:

- ◯ 8 weeks
- ◯ 12 weeks
- ◯ 24 weeks
- ◯ Other (please specify below)

Other duration of treatment regimen for non-cirrhotic patients with this genotype: __________________________________

Additional comments:

____

Sources for answers: __________________________________

5.2 DAA treatment eligibility

Is publicly funded direct-acting antiviral (DAA) treatment provided to chronic HCV patients in your country?

- ◯ Yes -- all patients with chronic HCV are eligible for publicly funded DAA treatment
- ◯ Yes -- patients who meet certain clinical criteria are eligible for publicly funded DAA treatment (please describe the criteria below)
- ◯ No -- DAA treatment is not publicly funded
- ◯ Do not know

Please describe clinical criteria required for patient eligibility for publicly funded DAA treatment: __________________________________

Additional comments:

____

Sources for answers: __________________________________

5.3 DAA treatment restrictions

In practice, what restrictions are there on access to direct-acting antivirals for the treatment of HCV infection in your country?

Please choose all answers that apply.

- ☐ None
- ☐ Fibrosis level: only patients above a certain fibrosis level are eligible for treatment
- ☐ Quotas: only a limited number of patients can be treated within a certain time period or a certain geographic area
- ☐ Alcohol use: people who currently drink alcohol are not treated
- ☐ Injecting drug use: people who injected drugs in the past are not treated, even if they are not currently injecting drugs
- ☐ Injecting drug use: people who are currently injecting drugs are not treated
- ☐ Injecting drug use: people who injected drugs in the past are only treated if they have abstained from injecting drugs for a specified period of time (please specify required abstinence time below)
- ☐ Injecting drug use: people who currently inject drugs or injected drugs in the past are only treated if they are receiving opioid substitution therapy
- ☐ Other restrictions (please describe below)
- ☐ Do not know

Please specify required abstinence time:

- ◯ At least 6 months
- ◯ At least 1 year
- ◯ At least 2 years
- ◯ Other

Other required abstinence time: ______________________________

Other restrictions, please specify (1): ______________________________

Other restrictions, please specify (2): ______________________________

Other restrictions, please specify (3): ______________________________

Additional comments:

____

Sources for answers: ______________________________

5.4 Number of people treated for HCV in 2015

Please indicate the total number of people who received HCV treatment in 2015: ______________________________

If this information is not available, please provide any recent official or estimated data, specifying the year: ______________________________

Additional comments:

____

Sources for answers: ______________________________

5.5 HCV treatment in non-hospital settings

Do any HCV patients in your country have the option of being treated in non-hospital settings? (Settings that are not within either inpatient or outpatient hospital facilities.)

◯ Yes
◯ No
◯ Do not know

What types of non-hospital settings?

Please choose all answers that apply.

☐ General practitioner clinics
☐ Addiction/opioid substitution clinics or harm reduction centres
☐ Other (please specify below)

Other type, please specify (1): ____________________

Other type, please specify (2): ____________________

Other type, please specify (3): ____________________

Additional comments:

____

Sources for answers: ____________________

5.6 HCV treatment geographic availability

Can HCV treatment be obtained from healthcare providers in all parts of your country?

◯ Yes
◯ No
◯ Do not know

Additional comments:

____

Sources for answers: ____________________

5.7 HCV treatment prison availability

Is HCV treatment provided in prisons in all parts of your country?

◯ Yes
◯ No
◯ Do not know

Additional comments:

____

Sources for answers: ____________________
